# Physiological and Transcriptomic Analysis of Two Types of Hami Melons in Low-Temperature Storage

**DOI:** 10.3390/plants14081153

**Published:** 2025-04-08

**Authors:** Wanqin Liao, Linlu Xiao, Xiangshuai Hao, Chunhui Shan, Zhongkai Zhou, Ming Ning, Fengxian Tang

**Affiliations:** 1 Engineering Research Center of Storage and Processing of Xinjiang Characteristic Fruits and Vegetables, Ministry of Education, School of Food Science, Shihezi University, Shihezi 832000, China; 2 Key Laboratory of Processing and Quality and Safety Control of Specialty Agricultural Products (Co-Construction by Ministry and Province), Ministry of Agriculture and Rural Affairs, School of Food Science, Shihezi University, Shihezi 832000, China; 3 Key Laboratory for Food Nutrition and Safety Control of Xinjiang Production and Construction Corps, School of Food Science, Shihezi University, Shihezi 832000, China

**Keywords:** Hami melon, low-temperature storage, physiological biochemistry, transcriptome

## Abstract

The Hami melon is a characteristic economic crop in Xinjiang. Long-term storage at low temperatures can cause cold damage and significantly impact the storage quality of Hami melon fruits. This study investigated the cold resistance of two Hami melon varieties under low temperatures, screened key genes, and further explored their resistance mechanisms. By comparing and analyzing the relationship between phenotypic morphology, physiological indicators, and storage time, it was found that the symptoms of cold damage in Hami melons are related to both storage time and variety. To analyze the response mechanisms of Hami melons to cold stress at the molecular level, we conducted transcriptome sequencing analysis on the cold-sensitive Hami melon variety Gold Queen and the cold-resistant variety Jia Shi. The analysis shows that cold stress induces the expression of these differentially expressed genes, which participate in the AsA-GSH cycling system, form the NADPH-P450 pathway, and establish the ERF-WRKY cold resistance pathway. This, in turn, increases the content of free proline in the fruits, clears denatured proteins within the fruit, maintains the stability of the redox system, and inhibits certain differentially expressed genes that regulate cell wall metabolism, thereby alleviating fruit softening and improving cold resistance.

## 1. Introduction

Hami melons (*Cucumis melo* var. *saccharinus*) are one of the horticultural specialties of Xinjiang, China, and one of its most important economic crops. Low-temperature storage is the most cost-effective and commonly used method for storing Hemi melon fruits and other produce. Low temperatures reduce fruit and vegetable respiratory rates and delay pathogenic microorganism growth; however, long-term low-temperature exposure can easily cause chilling injuries in stored items, with negative economic outcomes [1,2,3]. Hami melons are cold sensitive, and their long-term low-temperature storage may change their cell membrane permeability, increasing reactive oxygen species (ROS) production and accumulation, and affecting energy production and transmission. The resulting changes to the cell wall structure and physiological processes can cause chilling injury symptoms such as dehydration, crumpling, indentation, and browning, ultimately leading to fruit rotting [4,5,6,7].

Low temperatures and other adverse conditions, including high salinity and drought, cause oxidative stress in plants. The resulting accumulation of ROS activates the plant cell stress response, which involves enzymatic and non-enzymatic antioxidant defense systems that synergistically remove oxygen radicals and maintain cellular homeostasis and balance [8]. The enzymatic antioxidant defense includes catalase (CAT), glutathione peroxidase (GSH-Px), and superoxide dismutase (SOD), which catalyze the reduction of O^2−^ to H_2_O_2_ and its conversion to H_2_O and O_2_ [8,9,10]. Proline is a multi-functional amino acid that can eliminate excessive ROS in cells, and its massive accumulation is a common adaptive response to cold stress in plants [11,12]. Pyrroline-5-carboxylic acid synthetase (P5CS) and proline dehydrogenase (ProDH) catalyze the synthesis and degradation of proline, respectively, and play important roles in maintaining cellular free proline content and osmotic balance [13]. Bi et al. found a significant positive correlation between the proline accumulation level and chilling damage index under low temperature stress in melon [14]. Bokhary et al. observed that, in zucchini (*Cucurbita pepo* L.), a hot water treatment reduced ProDH activity, increased the free proline content, and significantly reduced the probability of CI, improving its cold resistance [15]. Furthermore, Chen et al. reported that cold stress caused a reduction in the free proline content in olive fruit by increasing ProDH activity and decreasing that of P5CS, resulting in chilling injury [16].

Adverse environmental conditions activate the expression of stress response genes through a series of complex signaling pathways, ensuring that the appropriate physiological and biochemical changes occur downstream [17]. Therefore, it is common to use transcriptomics to study stress response-related gene expression. Zhang et al. demonstrated that cold stress altered *AMY* and *BMY* expression in Hami melon fruits, which promoted starch degradation and increased soluble sugar levels, enhancing cold resistance [18]. Similarly, changes in the expression of *MYB76*, *ZAT*, and *AP2*/*ERF* enhanced the cold resistance of zucchini [19]. Transcriptomics has also been applied to the study of cold responses in fruits such as tomato (Solanum lycopersicum) [20], apricot (*P. armeniaca* cv.) [21], bayberry (*Myrica rubra*) [22], blueberry (*Vaccinium corymbosum* ‘Duke’) [23], and papaya (*Carica papaya* L.) [24]. In Jia Shi melons, a cold-tolerant variety, cold stress induced the expression of early response genes (*ICE1*, *CDPK*), late response genes (auxin response factor, *MYB*, *HSPs*, and *FAD*), and sugar transport protein [25].

In this study, we investigated the physiological and transcriptional responses of cold-sensitive (Gold Queen) and cold-resistant (Jia Shi) Hami melon varieties to cold storage stress. We identified the key differentially expressed genes (DEGs) related to cold resistance in Hami melons and connected them to the observed changes in the physiological parameters. This study provides theoretical references for further understanding the molecular mechanisms of cold resistance and shelf-life extension in post-harvest Hami melons.

## 2. Results

### 2.1. Physiological Characteristics of Cold-Sensitive and Cold-Tolerant Hami Melon Fruits

#### 2.1.1. Chilling Injury (CI) Symptoms Under Cold Storage Stress

**Figure 1:**
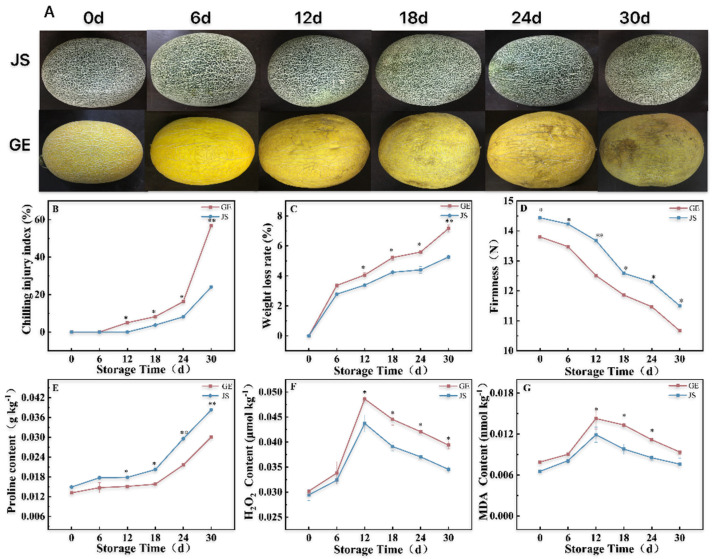
Fruit phenotypes and physiological indexes in Hami melon varieties under cold storage stress. (**A**) The changes in phenotype, (**B**) CI index, (**C**) weight loss rate, (**D**) hardness, (**E**) free proline content, (**F**) H_2_O_2_ content, (**G**) and MDA content of Hami melons. Error bars represent the standard deviation of the means. At the same storage time, * indicates significant differences (*p* < 0.05). ** = the number indicates a highly significant difference (*p* < 0.01).

In the early stage of storage (0–6 d) at low temperatures, the fruits of both varieties were healthy and plump, with no symptoms of CI (Figure 1A). Subsequently, surface pitting started to appear after 12 days in the cold-sensitive (GE) fruits, while it appeared after 18 days of storage in the cold-resistant (JS) variety. In the mid-stage period of cold storage (18–24 d), the CI of the two varieties increased dramatically (Figure 1B), which coincided with the deepening of fruit depressions and browning. After 30 days of storage, the CI of GE was 2.36 times higher than that of JS (*p* < 0.01).

#### 2.1.2. Weight Loss Rates Under Cold Storage Stress

For 0–6 days of cold storage, the weight loss rates were not significantly different between the two tested melon varieties, with decreases of approximately 3%. Longer storage periods increased the weight loss rates rapidly, which reached 7.16% and 5.26% after 30 d, for cold-sensitive and cold-tolerant melon varieties, respectively. The weight loss rate was 1.20 times higher in GE than in JS at 12 days (*p* < 0.05), when CI became apparent for the cold-sensitive variety, and higher for all longer storage periods (Figure 1C).

#### 2.1.3. Firmness Decreases Under Cold Storage Stress

The firmness of Hami melons was higher for both varieties before the cold storage period began and continuously decreased with the extension of storage time. Nevertheless, JS had a higher firmness than GE for all time points analyzed. The firmness of GE decreased from 13.48 ± 0.014 N to 12.51 ± 0.0118 N (7.20%) between day 6 and day 12, and that of JS from 13.68 ± 0.017 N to 12.59 ± 0.0331 N from day 12 to day 18, with a decrease of about 7.97%. These decreases coincided with the occurrence of CI in each variety (*p* < 0.05) (Figure 1D).

#### 2.1.4. Free Proline Under Cold Storage Stress

Overall, the free proline content increased with the cold storage period in both melon varieties, with JS having higher values than GE (*p* < 0.05) (Figure 1E). In early and mid-stage storage (0–18 d), the free proline content increased from 0.01315 ± 0.00062 to 0.01578 ± 0.00043 g kg^−1^ in GE and from 0.01491 ± 0.000172 to 0.0202 ± 0.00089 g·kg^−1^ in JS. Notably, a strong increase in the free proline content (13.23%) from day 12 to day 18 coincided with the appearance of chilling injuries and a significant decrease in firmness in JS (*p* < 0.05).

#### 2.1.5. H_2_O_2_ and MDA Contents Under Cold Storage Stress

In the early stage of storage (0–6 d), the H_2_O_2_ and MDA contents slowly increased for both melon varieties (Figure 1F,G) and peaked at 12 d. H_2_O_2_ reached 0.04861 ± 0.00054 and 0.04372 ± 0.00168 μmol kg^−1^ in GE and JS from day 6 to day 12, respectively, with values 60.91% and 48.51% higher than those registered on day 0 of storage. For MDA, the corresponding values were 0.01428 ± 0.00153 and 0.01191 ± 0.00111 nmol kg^−1^, with increases of 80.53% and 82.11% relative to 0 d. The increase in H_2_O_2_ and MDA coincided with CI onset and the decrease in firmness. Throughout the storage period, the H_2_O_2_ and MDA contents followed similar trends and were consistently higher in JS, the cold-tolerant variety (Figure 1F,G).

### 2.2. Enzymatic Activity in Hami Melon Fruits Under Cold Storage Stress

**Figure 2:**
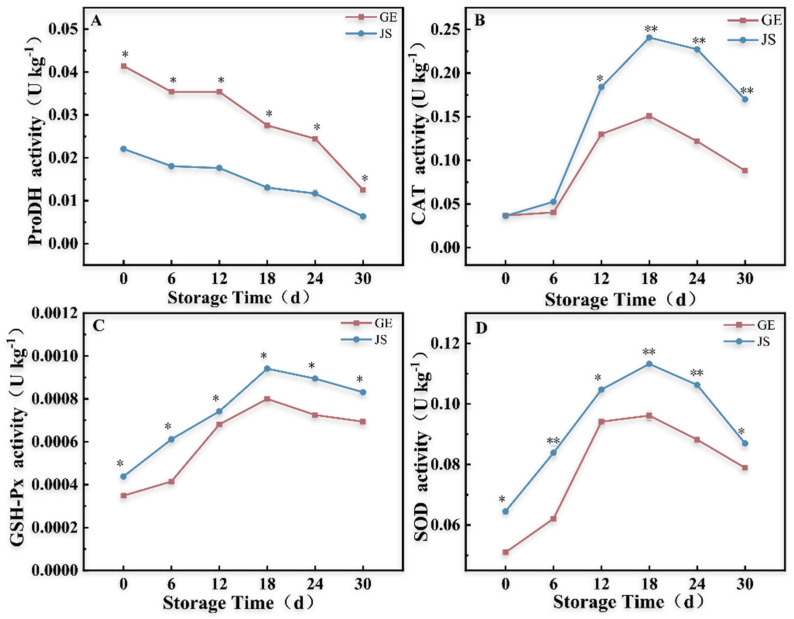
Enzyme activity in Hami melon fruits under cold storage stress. (**A**) ProDH activity, (**B**) CAT activity, (**C**) GSH Px activity, and (**D**) SOD activity. Error bars represent the standard deviation of the means. At the same storage time, * indicates significant differences (*p* < 0.05). ** = the number indicates a highly significant difference (*p* < 0.01).

For the totality of the storage period, ProDH activity decreased in melon fruits stored at low temperatures (Figure 2A). In GE, ProDH activity decreased from 0.04142 ± 0.00005 U kg^−1^ on day 0 to 0.01249 ± 0.00015 U kg^−1^ on day 30, whereas it decreased from 0.02207 ± 0.00026 U kg^−1^ on day 0 to 0.00634 ± 0.00009 U kg^−1^ on day 30 in JS. Throughout the storage period, the ProDH activity was lower in the cold-tolerant JS than in the melons of the GE cold-sensitive variety (*p* < 0.05).

The overall trend of anti-oxidase activity (CAT, GSH-Px, and SOD) was similar in both varieties, with an initial increase (0–18 d) and then a decrease as cold storage progressed (until day 30). CAT activity remained unchanged during the early stage of storage (0–6 d) (Figure 2C). On day 18, the CAT activity peaked for both varieties, with JS having a value 60% higher than GE (0.15083 ± 0.0005 and 0.24080 ± 0.00045 U kg^−1^, (*p* < 0.01)). Subsequently, the CAT activity decreased rapidly for both varieties. The GSH-Px activity gradually increased with the progression of storage time. In JS, GSH-Px activity increased rapidly from day 12 to day 18, with the highest value on day 18 (0.00094 ± 0.0000012 U kg^−1^). In GE, the quick increase from day 6 to day 12 was followed by the maximum achieved on day 18 (0.00080 ± 0.00000435 U kg^−1^) (Figure 2C). The SOD activity followed the same trend as that of the other enzymes peaking on day 18, followed by a rapid decline (*p* < 0.01) (Figure 2D). Throughout the storage period, JS had higher CAT, GSH-Px, and SOD activity levels than the GE cold-sensitive variety (*p* < 0.05).

### 2.3. Transcriptional Analysis of Hami Melon Varieties Under Cold Storage Stress

**Figure A1:**
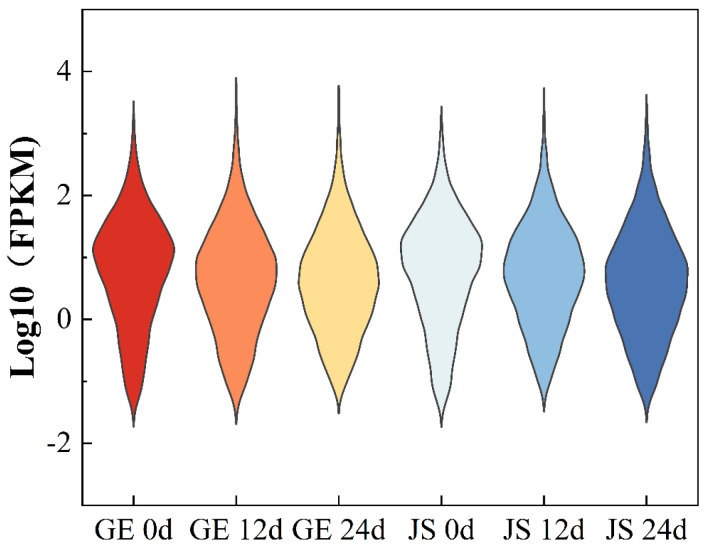
Gene expression level map of Hami melons under cold stress.

To further explore the effects of cold storage stress on Hami melons and the differences between varieties with different sensitivities, we performed a transcriptomic analysis via RNA-Seq, on the GE and JS. The sequencing data were filtered to obtain high-quality Clean Reads of approximately 1205 Mp per sample, with Q20 (%) and Q30 (%) greater than 97% and 90%, respectively (Table A2). The gene expression distribution was performed based on the FPKM of each gene. The distribution of the gene expression levels was discrete and uniform in the six sample groups of each variety and storage duration analyzed (Gold Queen melons and Jia Shi melons at 0, 12, and 24 d), and the overall gene expression richness in different samples was appropriate (Figure A1), which indicated that the quality of the transcriptome sequencing was high and that the data could be used for further analysis.

### 2.4. Analysis of DEGs in Cold-Stored Hami Melons

**Figure 3:**
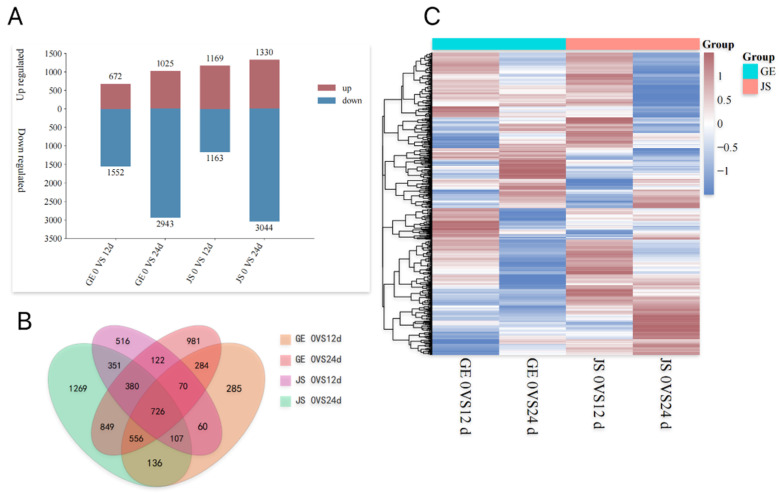
Analysis of DEGs in Hami melon fruits under cold storage stress. (**A**) Differential gene upregulation and downregulation analysis, (**B**) differential gene co-expression analysis, and (**C**) differential co-expression gene clustering analysis.

For GE, we identified a total of 2224 (672 upregulated and 1552 downregulated) and 3969 (1025 upregulated and 2943 downregulated) DEGs for day 12 and day 24 relative to day 0. In JS, there were 2332 (1169 upregulated and 1163 downregulated) and 4372 (1330 upregulated and 3044 downregulated) DEGs, respectively, for the same comparisons (Figure 3A). The number of DEGs was higher in the JS samples than in the GE ones, with 1.47 times more upregulated DEGs identified in the cold-tolerant variety.

To further compare the co-expressed DEGs in each experimental group, we plotted a Venn diagram (Figure 3B). A total of 726 DEGs were co-expressed in the four groups, suggesting that the response of Hami melons to cold stress was regulated by multiple genes with the progression of storage time. These co-expressed DEGs may relate to differentially expressed proteins (DEPs) that potentially respond to the CI occurring in Hami melons. Cluster analysis of co-expressed DEGs (Figure 3C) showed that JS responded to cold stress by upregulating more DEGs, and with the prolongation of storage time (24 d), the upregulation of co-expressed DEGs increased significantly in this variety.

### 2.5. GO and KEGG Analyses of Co-Expressed DEGs in Hami Melons Under Cold Storage Stress

**Figure 4:**
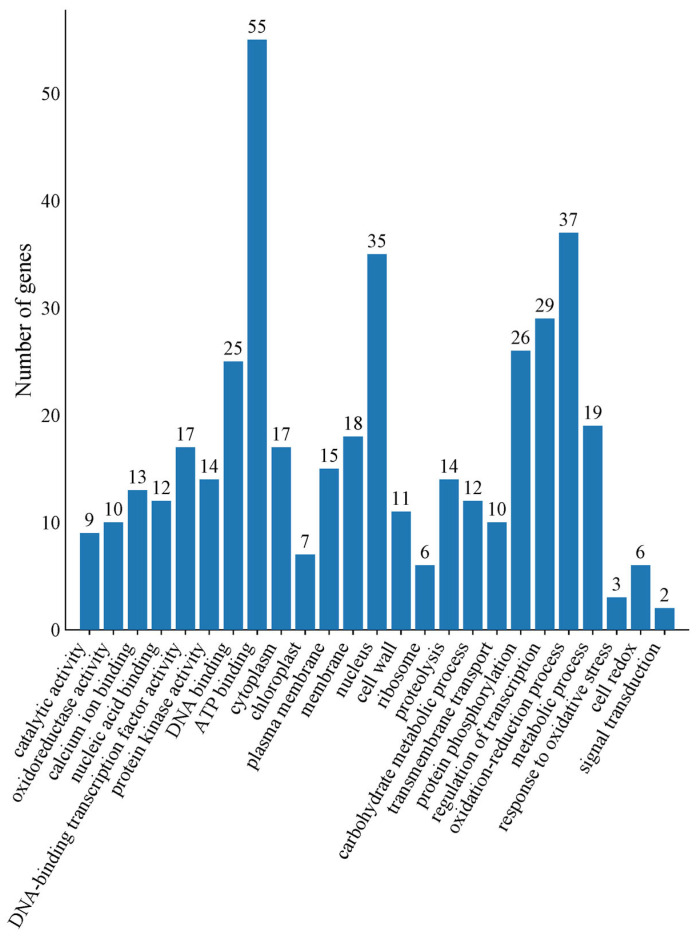
GO annotation of co-expressed DEGs in Hami melons under cold storage stress.

To further analyze the biological functions of the 726 co-expressed DEGs, we performed GO and KEGG functional annotation analyses. The GO enrichment analysis classified the co-expressed DGEs into molecular function (MF), cellular component (CC), and biological process (BP) groups (Figure 4). Cold storage stress mainly affected the oxidoreductase activity, calciumion binding, DNA-binding transcription factor activity, and ATP binding in the MF group. Based on the CC results, the DEGs were mainly located in the nucleus, cytoplasm, plasma membrane, and cell wall metabolism. For the BP group, they were mainly involved in the oxidation–reduction process, carbohydrate metabolic process, transmembrane transport, regulation of transcription, metabolic process, and response to oxidative stress.

**Figure 5:**
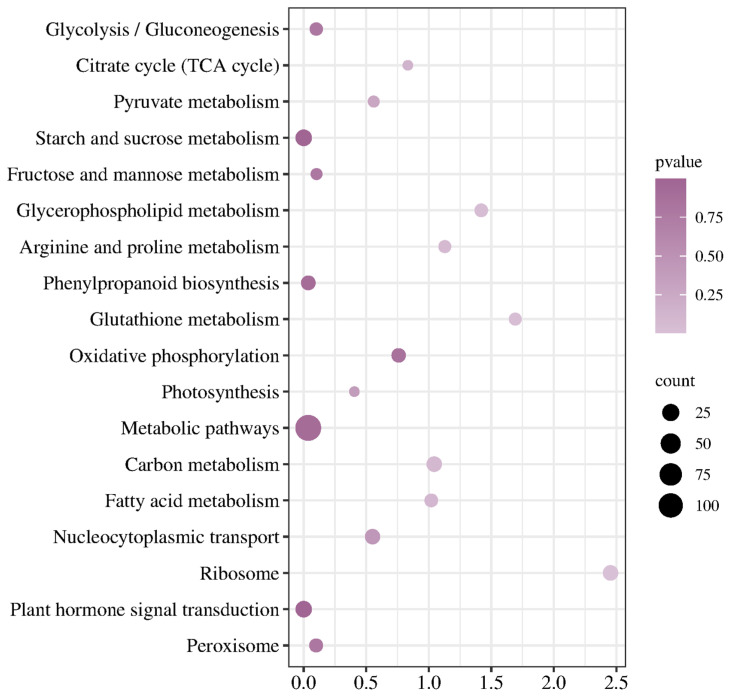
KEGG annotation of co-expressed DEGs in Hami melon fruits under cold storage stress.

KEGG enrichment analysis showed that co-expressed DEGs were mainly enriched in metabolic pathways, starch and sucrose metabolism, carbon metabolism, glycolysis/gluconeogenesis, phenylpropanoid biosynthesis, plant hormone signal transduction, arginine and proline metabolism, oxidative phosphorylation and nucleocytoplasmic transport, glutathione metabolism, and other metabolic pathways (Figure 5).

### 2.6. Analysis of Key Co-Expressed DEGs Related to the Cold Stress Response

To comprehensively investigate the transcriptional response to cold storage stress in Hami melons, 726 co-expressed DEGs were analyzed to identify those related to cold stress (Table 1). For the oxidoreductase activity group, the expression levels of three peroxidase genes (XM_008442029.1, XM_008460995.1, and XM_008467715.2), a *GAPD* gene (XM_008441186.2), a *CAT* gene (XM_008454735.2), a phenylalanine ammonia-lyase (*PAL*) gene (XM_008452678.2), a *HsF* gene (XM_008442056.2), a *HsP* gene (XM_008441060.2), a *P5CS* gene (XM_008444288.2), and a *CAT* gene (XM_008454735.2) were consistent with their enzymatic activity levels (Figure 2B). *PAL* (XM_008452678.2) was induced during the early and mid-stage storage period (12 d), and its expression decreased as the storage time increased. *HsF* (XM_008442056.2) and *HsP* (XM_008441060.2) expression increased with storage time and was higher in JS (*p* < 0.05). Notably, the expression of *P5CS* (XM_008444288.2) was consistent with the free proline content (Figure 1E).

For the carbohydrate metabolic process category, the expression levels of *trehalase* (XM_008439170.2), pectinesterase (*PE*) (XM_008463183.2), polygalacturonase (*PG*) (XM_008460083.2), two Galacturonosyl transferase (*GAUT*) genes (XM_008448052.2 and XM_008461151.2), and a *GAPDH gene* (XM_008441186.2) were similar to those of the *PAL* gene (XM_008452678.2). The *trehalase* gene (XM_008439170.2) reached its peak on day 12 in GE and then began to decline, whereas in JS, it increased gradually with the storage time. The expression of *PE* (XM_008463183.2), both *PG* genes (XM_017045662.1 and XM_008460083.2), and *GAUT* (XM_008448052.2) increased with the storage period, with lower values in JS.

Several DEGs related to cell wall metabolism were identified, including Xyloglucan endotransglycosylase/hydrolase (*XTHs*) genes (XM_008439950.2, XM_008443187.2, and XM_008467178.2), the *expansin* gene (XM_008461106.2), cytochromeP450 (*CYP450*) genes (XM_008466543.2, XM_017044466.1, XM_008439072.2, and XM_017044655.1), and *NAD(P)H* gene (XM_008441679.2). Notably, the expression of *XTHs* and *expansin* genes in GE was higher than in JS, whereas *CYP450* and *NAD(P)H* were lower in GE. Interestingly, *CYP450* genes (XM_008466543.2, XM_017044466.1, XM_008439072.2, and XM_017044655.1) and the *NAD(P)H* gene (XM_008441679.2) are also involved in the carbohydrate metabolic process.

Nine co-expressed DEGs, including four *ERF* genes (XM_008442055.2, NM_001319315.1, XM_008457109.2, and XM_008457900.2), a *WRKY* gene (XM_008466930.2), two NAC genes (XM_008468514.2, XM_008456444.2), and two *MYB* genes (XM_008450150.2 and XM_017046406.1) are involved in transcription regulation. The expression levels of *MYB* genes (XM_008450150.2 and XM_017046406.1), *NAC* genes (XM_008468514.2 and XM_008456444.2), and a *WRKY* gene (XM_008466930.2) were significantly higher in JS than in GE, with expression increasing with storage time.

### 2.7. qRT-PCR Confirmation of DEGs

**Figure 6:**
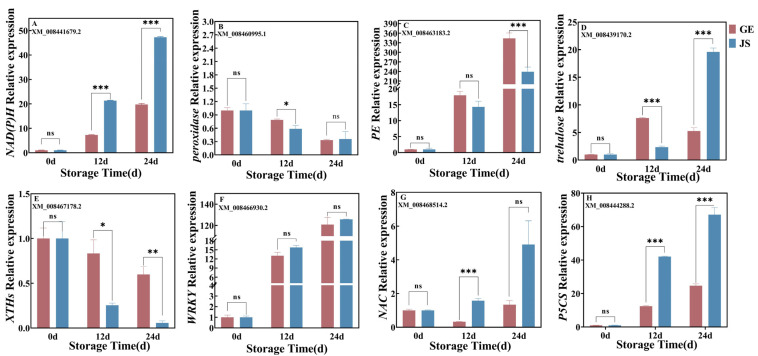
Relative mRNA levels of ten DEGs determined via quantitative qRT-PCR analysis. (**A**) NAD(P)H ubiquinone oxidoreductase, (**B**) peroxidase gene, (**C**) *PE* gene, (**D**) *trehalase* gene, (**E**) *XTHs* gene, (**F**) *WRKY* gene, (**G**) *NAC* gene, and (**H**) *P5CS* gene. Error bars represent the standard deviation of the means. At the same storage time, ns indicates no difference. * indicates significant differences (*p* < 0.05). ** = the number indicates a highly significant difference (*p* < 0.01). *** = the number indicates a significant difference (*p* < 0.001).

To verify the accuracy of the RNA-Seq results, eight key co-expressed DEGs under cold storage stress were randomly selected for qRT-PCR analysis: *NAD(P)H* (XM_008441679.2), peroxidase (XM_008460995.1), *PE* (XM_008463183.2), *trehalase* (XM_008439170.2), *XTHs* (XM_008467178.2), *WRKY* (XM_008466930.2), *NAC* (XM_008468514.2), and *P5CS* (XM_008444288.2). qRT-PCR quantification confirmed the RNA-seq sequencing results (Figure 6).

## 3. Discussion

### 3.1. Physiological Changes in Hami Melon Varieties Under Cold Storage Stress

Fruits and vegetables stored at cold temperatures for prolonged post-harvest periods undergo a series of physiological changes that directly affect their storage life and commercial value. Low-temperature storage is one of the most economical and effective methods to prevent fruit and vegetable decay. However, under unsuitable low-temperature storage conditions, CI may occur on the fruit surface, leading to depression, browning, water immersion, and other symptoms that decrease the product’s value [7,26]. Our results confirm these negative effects of prolonged cold storage (Figure 1A). Additionally, we observed a decrease in the water content coinciding with the occurrence of CI. With longer cold storage periods, the weight loss rate of fruit increased, and fruit crumpling and depression intensified. Therefore, reducing water loss is an effective means to prevent weight loss and CI aggravation in fruit [27]. In this study, the onset of CI occurred earlier in GE than in its JS counterparts, and the weight loss rate and CI were also higher in the cold-sensitive GE variety (Figure 1A–C), which is consistent with previous findings [28].

ROS are an electron-reduction product of oxygen and include superoxide anion, hydrogen peroxide, hydroxyl radical, and other peroxides [29]. Cold stress leads to the production and accumulation of ROS in cells, and excessive ROS can disrupt normal metabolism by oxidizing lipids, damaging cell membranes, and oxidizing proteins, resulting in apoptosis [25]. The main ROS markers in plants are H_2_O_2_ and MDA, whose accumulation is closely related to fruit cell membrane oxidation. We showed that the over-accumulation of H_2_O_2_ and MDA in Hami melons stored under cold stress (Figure 1F,G) led to a decrease in the cell membrane osmotic capacity and an increase in membrane damage, resulting in a higher weight loss rate (Figure 1C) and a lower firmness (Figure 1D). The aggravation of CI ultimately led to fruit decay (Figure 1A). Compared with cold-sensitive GE, JS had a delayed onset of CI and an extended shelf life by maintaining low H_2_O_2_ and MDA contents and high firmness (Figure 1A,D,F,G).

### 3.2. Enzymatic Activity Changes in Hami Melons Under Cold Storage Stress

Proline is a well-known organic osmotic regulator, with important roles in stress responses in fruits and vegetables, particularly in responses to cold stress [30]. ProDH is the rate-limiting enzyme in the proline degradation pathway, catalyzing the production of pyrroline-5-carboxylic acid (P5C) from proline, whereas P5CS is the key enzyme that catalyzes the synthesis of proline. These enzymes work together to maintain the osmotic regulation balance of the plasma membrane in response to stress [31,32,33]. Our analyses showed that the free proline contents increased rapidly in JS and reached higher values than for GE under cold stress storage (Figure 1E). The ProDH activity decreased gradually with the prolongation of storage time and was lower in JS (Figure 2A). Notably, prolonged storage duration led to progressive elevation in the fruit weight loss rates, with GE displaying a significantly higher mass reduction compared to its JS counterparts (*p* < 0.05; Figure 1C). Subsequent transcriptional profiling demonstrated the storage time-dependent upregulation of the *P5CS* gene (XM_008444288.2), wherein JS exhibited markedly elevated expression levels relative to GE (*p* < 0.05; Table 1). Therefore, we hypothesized that Hami melons enhanced cold resistance through the following synergistic mechanisms: suppressing proline degradation via the inhibition of ProDH activity and enhancing proline biosynthesis through the upregulation of *P5CS* gene expression, which collectively elevated free proline accumulation in fruits, ultimately conferring stronger low-temperature resistance by reducing fruit weight loss rates during storage. These results are consistent with those obtained in banana (*Musa* spp., AAA group cv. “Brazil”) [34] and zucchini [35] under adverse conditions.

Plant anti-oxidases play a key role in ROS scavenging. SOD can rapidly reduce superoxide to H_2_O_2_ under stress conditions, and then CAT and GSH-Px further convert H_2_O_2_ to H_2_O to scavenge the accumulation of H_2_O_2_ [36]. The AsA-GSH cycle is another important ROS scavenging system. It uses ROS as a substrate to generate water-soluble antioxidants (ascorbic acid) and maintain the intracellular redox dynamic balance in response to stress [37,38,39]. Yao et al. reported that an exogenous GSH treatment alleviated chilling injury during low-temperature storage by upregulating *CaDHAR1*, promoting the accumulation of AsA and GSH, and therefore, enhancing the antioxidant capacity of *Capsicum frutescens* L. (syn. *C. annuum* L.) var. grossum [40]. Székelyg et al. demonstrated that the accumulation of proline in the Arabidopsis *P5CS1* mutant was correlated with the stability of the GSH-AsA cycle and enzyme activity under stress [41]. Here, we showed that the GSH-Px content was significantly higher in JS than in GE under cold storage stress (Figure 2C). Notably, the GSH-Px activity increased abruptly in JS for fruits stored at cold temperature for 12–18 days, which coincided with the onset of CI symptoms (Figure 1B).

### 3.3. Impacts of Cold Storage Stress on Hami Melon Oxidoreductase Activity

Oxidative stress is a prevalent biological process during post-harvest fruit storage. When plants are subjected to cold stress in storage, ROS accumulate, resulting in oxidative stress, which in turn triggers cellular stress-related signals to scavenge ROS, including the increased production of antioxidants such as phenolic substances [42,43]. For example, *PAL* activity is closely related to chilling injury symptoms in fruits and vegetables [44]. Under cold stress, fresh walnuts (*Juglans regia* L.) enhance their cold resistance by promoting *PAL* activity to increase the soluble phenolic content [45]. In banana fruits, heat treatments upregulate *PAL* at the transcriptional and translational levels, enhancing the subsequent cold resistance [46]. In this study, *PAL* (XM_008452678.2) expression was induced in both Hami melon varieties in the early and mid-stages of storage (12 d), and the expression was higher in JS than in GE.

The stress protein (HsP) is produced by plants during special periods of adversity or development and protects cells through molecular chaperone mechanisms that increase resistance to adverse conditions [47]. Cold stress significantly induces *HsP* expression in potatoes, which reduces solute leakage and clears denatured proteins, improving their overall cold resistance [48]. Heat-treated peach fruits (*Prunus persica* (L.) Batsch) enhance cold stress resistance by increasing antioxidant activity through higher *PpHsPs* expression, which maintains the dynamic balance of ROS [49]. Furthermore, the heat shock factor (*HsF*) can also respond to stress by binding the HSE element of *HsP* genes to form a heat stress transcription factor [50,51,52]. In this study, *HsF* (XM_008442056.2) and *HsP* (XM_008441060.2) were progressively upregulated in both melon varieties under cold storage, with higher expression observed in JS at the mid–late stage (24 d). Hence, Jia Shi melons’ higher cold resistance might result from a relatively intact plasma membrane in the early and mid-stage of storage, less protein damage, and lower *HsP* expression. With the extension of storage time, the CI worsened, the plasma membrane was severely damaged, and proteins were polymerized and denatured. Thus, we conclude that *HsP* expression increased to remove excessive ROS and degrade denatured proteins in response to low-temperature stress.

### 3.4. Effects of Cold Storage Stress on Carbohydrate Metabolic Processes in Hami Melons

The carbohydrate metabolic process is fundamental in organisms and a key factor in regulating low-temperature adaptation in plants [53]. D-Trehalose anhydrous is a disaccharide and an important stress protector that plays a positive role in low-temperature and salt stress responses [54]. Through low-temperature metabolomic profiling using nuclear magnetic resonance (NMR) spectroscopy, Wang et al. demonstrated that exogenous trehalose application not only enhances sucrose biosynthesis in post-harvest peach fruits, but also effectively mitigates chilling injury symptoms during cold storage [55]. Liu et al. demonstrated that exogenous D-Trehalose anhydrous treatments in melon seedlings could significantly increase SOD, GSSG, and CAT activities, reduce the relative conductance of melon leaves, and enhance cold stress resistance [56]. In this study, with the extension of storage time, GE decayed severely (24 d), and the expression of *trehalase* (XM_008439170.2) decreased. Conversely, JS improved its cold resistance by increasing the expression of *trehalase* (XM_008439170.2) throughout storage and prolonging its expression period. We hypothesize that during the early-to-mid-storage phase (12 d), cold-sensitive GE upregulate the expression of the *trehalase* gene (XM_008439170.2), thereby promoting sucrose biosynthesis and accumulation. Simultaneously, this upregulation enhances antioxidant enzyme activities (e.g., SOD, CAT) to mitigate chilling stress. However, in the late storage phase (24 d), the expression of *trehalase* (XM_008439170.2) is significantly suppressed in GE genotypes, leading to exacerbated fruit decay. In contrast, the cold-tolerant JS genotype sustains the upregulation of *trehalase* (XM_008439170.2), which ensures stable sucrose accumulation and maintains higher antioxidant enzyme activity, ultimately conferring enhanced cold resistance.

Pectin is a structurally complex polysaccharide that is commonly associated with fruit softening, which in turn is closely related to PE and PG activity [57]. In this study, *PE* (XM_008463183.2) and *PG* (XM_017045662.1 and XM_008460083.2) expression levels increased with the storage time, reaching higher levels in GE. Therefore, we propose that cold-resistant JS alleviate pectin degradation by suppressing the expression of *PE* and *PG* genes to maintain fruit firmness and improve cold resistance, which is consistent with previous findings [18].

### 3.5. Effects of Cold Storage Stress on Cell Wall Metabolism in Hami Melon Fruits

The cell wall plays an important role in cold stress resistance in fruit by forming a protective barrier to the environment [58,59]. It is a complex structure mainly composed of cellulose, hemicellulose, pectin, and lignin, and is also a dynamic structure with spatial and temporal variability during cell synthesis, degradation, and reorganization [60]. Cytochrome P450 (CYP450) is a superfamily of heme-based enzymes ubiquitous in organisms, widely involved in cell wall metabolism and adverse stress response, among other important biological processes [61,62]. Under low-temperature stress, Arabidopsis enhances lignin synthesis to promote cold resistance by regulating the phenylpropanoid–lignin synthesis pathway, which consists of *CYP450* and *NADPH* [63,64]. In this study, *CYP450* (XM_008466543.2) and *NAD(P)H* (XM_008441679.2) expression increased with the storage time. Their transcript levels were higher in JS than in GE. This indicates that the NADPH-P450 pathway also exists in Hami melons under cold storage stress, conferring cold resistance by participating in phenylpropanoid–lignin biosynthesis.

*XTHs* (XM_008443187.2) and *expansin* (XM_008461106.2) are mainly involved in cell wall modification and are key regulators in fruit maturation softening and cell wall reconstruction [65]. Han et al. found that the overexpression of *DkXTH8* in tomatoes altered their cell wall structure, causing fruit softening [66]. Carvajal et al. demonstrated that zucchini expansins accelerated fruit softening by participating in the cell wall metabolism process, which was positively correlated with the occurrence of CI under cold stress [67]. In this study, cold storage stress promoted *XTHs* (XM_008443187.2) and *expansin* (XM_008461106.2) expression in both varieties, but GE had higher transcript levels than JS with longer storage time, leading to the accelerated softening of fruit and early onset of CI symptoms (Figure 1A,B,D) in the cold-sensitive variety.

### 3.6. Effects of Cold Storage Stress on Transcriptional Regulation in Hami Melons

Transcription factors respond to abiotic stress by regulating the transcription levels of downstream target genes [68]. *ERF* and *WRKY* are specific transcription factors widely present in plants that have been extensively studied for their role in biological processes such as plant growth and development, signaling transduction, and stress response [69]. Sun et al. found that *VaERF092* regulated *VaWRKY33* by binding to GCC elements in downstream target genes, thereby enhancing cold resistance in grapes (*Vitis amurensis*) [69]. Hu et al. reported that *ERF15* improved cold resistance by activating *WRKY6* expression in tomato plants [70]. Qu et al. demonstrated that inducing *CiWRKY31* expression improved cold resistance in citrus fruits (*Citrus ichangensis*) [71]. In this study, three *ERF* (XM_008442055.2, NM_001319315.1, and XM_008457900.2) and one *WRKY* transcription factor (XM_008466930.2) were upregulated with cold storage time progression. Notably, their expression trends were similar throughout the storage period. Therefore, it is likely that Hami melons activate downstream *WRKY* expression by inducing *ERF* transcription to enhance their cold resistance.

The plant-specific transcription factor family, *NAC*, plays a key role in plant growth, development, and biotic and abiotic stress responses. Pears can be equipped with cold resistance by inducing the expression of *PbNAC1* to reduce intracellular ROS levels [72]. Under cold stress, banana fruits directly activate downstream target genes, *MaCESA6B* and *MaCESA7*, by upregulating *MaNAC1* to participate in the plant secondary cell wall metabolism [73]. In this study, two *NAC* transcription factors (XM_008468514.2 and XM_008456444.2) were upregulated under cold storage stress, and their expression was higher in JS. Additionally, two *MYB* transcription factors (XM_008450150.2 and XM_017046406.1) had significantly increased expression with the cold storage time, which is consistent with previous results [25].

## 4. Materials and Methods

### 4.1. Materials and Treatments

Using two varieties of Hami melons—Gold Queen Melons (GE) and Jia Shi Melons (JS)—promoted in Xinjiang, China as research materials, we conducted a study. Both varieties of Hami melons were collected from the 121st Regiment Farm in Shihezi, Xinjiang. The melons were oval in shape, uniform in size, and free from pests, diseases, and mechanical damage, with fresh stems and skins. In this study, two evenly sized Hami melons of each variety were sent to the School of Food Science at Shihezi University in Xinjiang within 12 h and stored in a cold storage facility maintained at a temperature of 0.5 °C (ranging from 0 ± 0.5 °C), with a relative humidity controlled between 75% and 85%. Samples were collected at 0, 6, 12, 18, 24, and 30 days of storage. The fruit skin was removed and the flesh was diced, frozen with liquid nitrogen, and stored in a −80 °C freezer for the measurement of various indicators. Each variety of fruit had three replicates, with each replicate consisting of three fruits.

### 4.2. Quantification of Physiological Parameters

The chilling injury (CI) of Hami melon fruits was determined after the different periods of storage at low temperatures. The symptoms of chilling injury observed included browning and the indentation of the fruit surface. Based on the intensity of the phenotypic changes, the fruits were assigned to four classes, as described before [6]. The CI was calculated using the following formula:

CI = ∑ (Chilling_injury_grade × fruit_number)/(n × N)

where n refers to the highest chilling injury index and N refers to the number of fruits analyzed.

The weight loss rate and firmness were measured using the methods described by Ning et al. [74]. The weight loss rate was determined as follows: weight loss rate (%) = (initial weight—current weight)/(initial weight) × 100%. To evaluate firmness, the melon fruits were cut longitudinally, and then transversely, into slices of approximately 1.5 cm in thickness. Samples from the equatorial region were then collected using a 1.6 cm diameter puncher and modified into 1 cm thick cylinders. Firmness was tested using an analyzer (SMSTA. XTplus, Stable Micro System, Godalming, UK) in the puncturing mode. Five repeats were performed for each fruit, and the average was calculated.

The proline content was determined according to Zuo et al. [35]. The sample tissue was ground with 3% sulfosali-cylic acid (2:5, *w*/*v*) and then placed in a boiling water bath for 5 min. After shaking for 10 min at 300 rpm/min, the homogenate was centrifuged at 12,000× *g* for 20 min. The assay mixture contained 2 mL of crude extraction, an equal volume of acid-ninhydrin reagent and glacial acetic acid, and 5 mL of toluene was added to partition after boiling for 30 min. Subsequently, the absorbance of the organic phase was determined at OD520. The proline content was obtained using a standard curve, and expressed as g proline per g fresh weight (g kg^−1^).

The H_2_O_2_ content was determined according to the method described by Carvajal et al. [19]. Briefly, the fruits were ground in liquid nitrogen and homogenized in 0.1% (*w*/*v*) TCA (1:4, *w*/*v*). The supernatant was collected via centrifugation at 4 °C and 12,000× *g* for 15 min. The reaction mixture consisted of 0.25 mL of supernatant, 0.25 mL of 100 mM potassium phosphate buffer (pH = 7), and 1 mL of 1 M KI. The reaction was color developed in the dark for 1 h and the absorbance was measured at 390 nm. The amount of H_2_O_2_ was calculated using a standard curve, and expressed as μmol kg^−1^.

The malondialdehyde (MDA) content was determined according to Heath and Packer [75], with slight modifications. Briefly, the exocarp was ground in liquid nitrogen and homogenized (1:4, *w*/*v*) in 20% (*w*/*v*) TCA, and butylated hydroxytoluene was added to reach a final concentration of 0.67%. The homogenate was centrifuged at 4 °C and 10,000× *g*. The supernatant was mixed with 0.5% (*w*/*v*) TBA in 20% TCA at a ratio of 1:4 (*v*/*v*). The mixture was heated in a 95 °C water bath for 30 min, immediately cooled in ice to stop the reaction, and centrifuged for 10 min (4 °C, 4000× *g*). The absorbances of supernatant at 532 and 600 nm were then measured, and the MDA content was calculated by subtracting the non-specific absorption at 600 nm from the absorption at 532 nm and using a standard curve. The results were expressed as nmol kg^−1^.

### 4.3. Measurement of Enzymatic Activity

A sample of 0.5 g of melon fruit was added to 1 mL of extraction solution and homogenized in an ice bath. The prepared homogenate was centrifuged at 4 °C (10,000× *g*, 30 min), and the supernatant was taken for analysis. The enzymatic activities were determined using the microplate assay kits for GSH-Px, CAT, SOD, and ProDH (Suzhou Grace Biotechnology Co., Ltd. (Suzhou, China)). The enzyme activity unit (U) was defined as the amount inducing 0.001 absorbance change per minute per gram of Hami melon under standardized conditions, with results expressed as U·kg⁻¹ following spectrophotometric assay protocols.

### 4.4. DEG Annotation and Functional Analysis

Total RNA extraction for each experimental group (variety and storage time of 0, 12, or 24 d) was performed using a plant total RNA extraction kit (Shanghai Yuanye Biotechnology Co., Ltd., Shanghai, China) following the manufacturer’s instructions. cDNA libraries were made for each sample and BGISEQ-500 transcriptome sequencing was subsequently performed (BGI, Shenzhen BGI Co., Ltd., Shenzhen, China). After filtering the raw reads, the output went through a quality control step. The gene expression quantification was carried out using RSEM version 1.3.1 [76], and the results were presented as FPKM for each gene.

The data from different samples were used for the identification of DEGs [77]. DEGs were analyzed according to Kim and van de Wiel [78], with slight modifications: genes with a FDR (False Discovery Rate) ≤ 0.001, Log2FC (fold change) ≥ 2 or ≤−2 were defined as DEGs.

Enrichment analysis of the DEGs was performed based on gene ontology (GO) (http://www.geneontology.org/, accessed on 17 March 2024) and the Kyoto Encyclopedia of Genes and Genomes’ (KEGG; https://www.kegg.jp/, accessed on 18 March 2024) descriptions based on their hypergeometric distribution. The significant levels of the terms and pathways were assessed with a Q value ≤ 0.05 via Bonferroni correction [79].

### 4.5. Analysis via qRT-PCR

Total RNA was extracted using a plant RNA kit (R33152, Shanghai, China), and cDNA was synthesized using a genome reverse transcription premix kit (CW2020M, Taizhou, China). A real-time quantitative reverse transcription polymerase chain reaction (qRT-PCR) was then performed using specific primers designed by Prime 6.0 software (Table A1). qRT-PCR was conducted using the SybrGreen qPCR Master Mix (Taizhou, China) and the Quant Studio Design and Analysis Software (Version 1.5.1) with three biological replicates. The qRT-PCR conditions were as follows: pre-denaturation at 95 °C for 3 s, denaturation at 95 °C for 5 s, and annealing/extension at 60 °C for 30 s, for a total of 45 cycles. Dissociation curve analysis was performed as follows: 15 s at 95 °C, 1 min at 60 °C, 15 s at 95 °C, and 30 s at 50 °C. Amplification was carried out in a 96-well plate, and the SybrGreen signal was detected at the end of each extension step at 60 °C in each cycle. Normalize all Ct values using the internal reference gene (GAPDH) and calculate the relative expression of each target gene using the 2^−ΔΔCt^ method.

### 4.6. Data Analysis

The data represent the mean ± standard deviation (SD) of three biological replicates. One-way analysis of variance (ANOVA) and Dunnett’s T tests (*p* = 0.05) were performed using Origin2022 and GraphPad Prism 9.5.0, with the results plotted in a graph. GO and KEGG annotation analysis, heatmaps, and Venn diagrams were generated using https://www.bioinformatics.com.cn (accessed on 13 March 2024), an online platform for data analysis and visualization. The mechanism diagrams were created using Figdraw 2.0.

## 5. Conclusions

**Figure 7:**
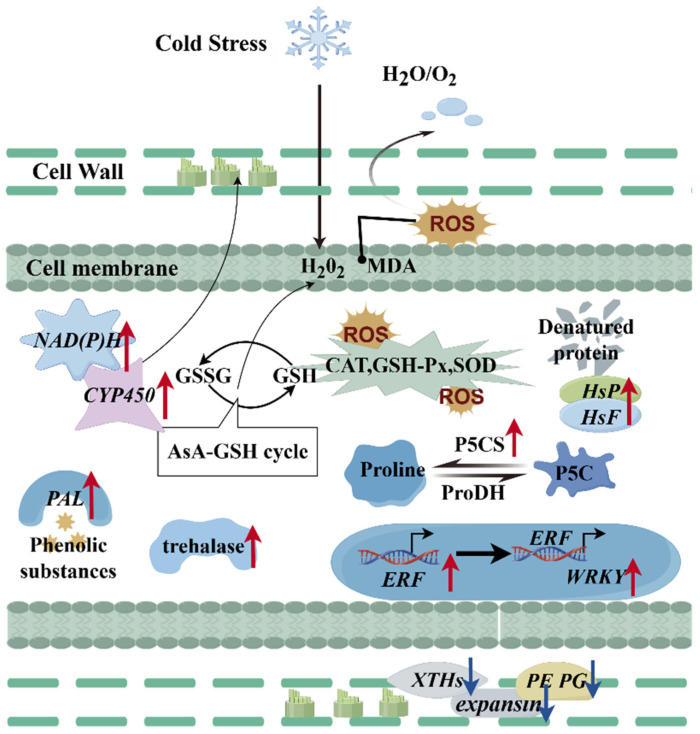
Model of stress response pathways in Hami melons under cold storage stress.

Based on integrated physiological, biochemical, and transcriptomic data, we propose a molecular mechanism underlying cold stress responses in Hami melons (Figure 7). When subjected to cold stress, membrane phase transitions occur, initiating cold signal transduction. This process elevates the cellular levels of osmoregulatory substances (H₂O₂, MDA) and reactive oxygen species (ROS), disrupting osmotic homeostasis. Consequently, the transcriptional activation of stress-responsive genes—including *trehalase*, *P5CS*, *CYP450*, *NAD(P)H*, *HSP*, *HSF*, *ERF*, and *WRKY*—orchestrates three coordinated mechanisms: engagement in the AsA-GSH cycle, the establishment of the NADPH-P450 pathway, and the formation of the ERF-WRKY cold resistance pathway. These synergistic interactions enhance free proline biosynthesis, amplify antioxidant enzyme activities, and facilitate denatured protein clearance. Concomitantly, the transcriptional repression of cell wall-modifying genes (*XTHs*, *expansin*, *PE*, and *PG*) mitigates pectin degradation, thereby reducing cell wall softening and preserving fruit firmness. The integrated regulation of these processes maintains redox equilibrium and metabolic stability, collectively conferring cold tolerance. This study provides mechanistic insights into cold resistance mechanisms in Hami melons, while also pinpointing key genetic components for cold adaptation, including *trehalase*, *P5CS*, and the synergistic ERF-WRKY transcriptional regulatory pathway. These findings establish essential molecular targets for developing cold-tolerant Hami melon cultivars through molecular breeding strategies and improving post-harvest storage performance.

## Figures and Tables

**Table 1:** List of some important differentially expressed genes in Hami melon fruits under cold stress.

Gene ID	Gene Name	Fold Change			
		GE 0.5 °C		JS 0.5 °C	
		0 vs. 12 d	0 vs. 24 d	0 vs. 12 d	0 vs. 24 d
Oxidoreductase activity					
XM_008442029.1	peroxidase	9.000	13.454	16.336	17.877
XM_008460995.1	peroxidase	0.067	0.0112	0.025	0.006
XM_008467715.2	peroxidase	10.196	21.407	11.551	15.562
XM_008441186.2	GAPD	9.254	10.556	5.657	8.225
XM_008454735.2	CATisozyme	46.527	243.8753	97.681	1136.199
XM_008452678.2	PAL	0.139	0.102	0.178	0.129
XM_008442056.2	HsF	7.726	12.188	7.829	33.612
XM_008441060.2	HsP	11.820	22.290	44.575	104.598
XM_008444288.2	P5CS	10.778	20.252	48.168	71.506
Carbohydrate metabolic process					
XM_008441186.2	GAPDH	9.254	10.556	5.657	8.225
XM_008439170.2	trehalase	8.456	5.063	5.242	10.339
XM_008448052.2	GAUT	5.540	14.621	4.595	13.929
XM_008463183.2	PE	53.817	333.144	36.504	142.025
Cell Wall metabolism					
XM_008439950.2	XTHs	29.041	51.268	11.472	6.821
XM_008443187.2	XTHs	32.223	106.153	22.814	39.947
XM_008466543.2	CYP450	21.259	157.586	93.054	1217.748
XM_017044655.1	CYP450	0.187	0.058	0.199	0.164
XM_017044466.1	CYP450	0.189	0.092	0.243	0.139
XM_008439072.2	CYP450	86.823	23.918	165.421	398.932
XM_008441679.2	NAD(P)H- ubiquinone oxidoreductase	9.849	18.001	24.933	48.5023
Regulation of transcription					
XM_008442055.2	ERF109	187.403	286.026	276.282	1595.729
NM_001319315.1	ERF071	18.000	20.112	5.134	8.574
XM_008457109.2	ERF054	10.853	23.752	33.128	24.933
XM_008457900.2	ERF109	36.002	46.851	37.271	155.417
XM_008466930.2	WRKY 40	24.084	105.420	24.420	117.148
XM_008468514.2	NAC 2	7.516	10.928	23.264	84.449
XM_008456444.2	NAC 72	3.706	4.627	5.502	6.543
XM_008450150.2	MYB 48	4.028	13.737	12.553	21.706
XM_017046406.1	MYB 44	88.035	192.672	3061.451	11,910.943

## Data Availability

Data are contained within the article.

## Abbreviations

The following abbreviations are used in this manuscript:

GE	Gold Queen Melons
JS	Jia Shi Melons
ROS	Reactive oxygen species
CAT	Catalase
GSH-Px	Glutathione peroxidase
SOD	Superoxide dismutase
ProDH	Proline dehydrogenase
P5CS	Pyrroline-5-carboxylic acid synthetase
DEGs	Differentially expressed genes
CI	Chilling injury
MDA	Malondialdehyde
GO	Gene ontology
KEGG	Kyoto Encyclopedia of Genes and Genomes
DEPs	Differentially expressed proteins
MF	Molecular function
CC	Cellular component
BP	Biological process
PAL	Phenylalanine ammonia-lyase
PE	Pectinesterase
PG	Polygalacturonase
GAUT	Galacturonosyl transferase
XTH	Xyloglucan endotransglycosylase/hydrolase
CYP450	CytochromeP450
HsF	Heat shock factor
HsP	Heat shock protein

## Appendix A

### Appendix A.1

**Table A1:** The sequences of specific primers used for RT-qPCR analysis.

Name	Accession Number	Forward Primer (5′–3′)	Reverse Primer (5′–3′)
*NAD(P)H-ubiquinone oxidoreductase*	XM_008441679.2	TGGCTCTGTCTTGAACCT	CTGTGGCGTTATACTTATTCC
*peroxidase*	XM_008460995.1	CGAGAATGGTTAGAGAATACAG	ATTAACAACGCCACATTGC
*PE*	XM_008463183.2	CGAGGACAAGGAGTAGCA	TCATAGAAGGATCTTCCATAGC
*trehalase*	XM_008439170.2	CTTGAGCGTCTTCAGGTT	ACAGAGCCATTGGAGGAT
*XTHs*	XM_008467178.2	ACCAGCCGTTCGTATCAAAGTACC	ACCCACTCCATTGCCTTGTATTGC
*WRKY*	XM_008466930.2	AAGGTGAACACAATCATCCA	GCTTCCATAATCGGTTTCG
*NAC*	XM_008468514.2	CGATGTCAGATGGCAATCA	CTCCGAACCGCTTGAATC
*P5CS*	XM_008444288.2	CATACGAGGATTCTTCTGGTA	ACAAGCCTTCAACATCACTA

### Appendix A.2

**Table A2:** Summary of sequencing data.

Sample	Raw Data Size (Mp)	Clean Data Size (Mp)	Clean Data Rate (%)	Clean Read Q20 (%) ≥ 90	Clean Read Q30 (%) ≥ 90
GE 0.5 °C-0 d	1206.83	1205.51	99.89	97.9 (Y)	91.14 (Y)
GE 0.5 °C-12 d	1206.81	1205.52	99.89	97.8 (Y)	90.98 (Y)
GE 0.5 °C-24 d	1206.82	1204.60	99.81	97.6 (Y)	91.01 (Y)
JS 0.5 °C-0 d	1206.83	1204.93	99.84	97.9 (Y)	91.54 (Y)
JS 0.5 °C-12 d	1206.81	1205.48	99.88	97.9 (Y)	91.21 (Y)
JS 0.5 °C-24 d	1206.82	1204.82	99.83	97.9 (Y)	92.02 (Y)

Note: ‘Y’ in the table indicates passing this quality control, while ‘N’ indicates not passing.
